# High gamma-glutamyl hydrolase and low folylpolyglutamate synthetase expression as prognostic biomarkers in patients with locally advanced gastric cancer who were administrated postoperative adjuvant chemotherapy with S-1

**DOI:** 10.1007/s00432-019-03087-8

**Published:** 2019-11-21

**Authors:** Yukio Maezawa, Kentaro Sakamaki, Naohide Oue, Yayoi Kimura, Itaru Hashimoto, Kentaro Hara, Kazuki Kano, Toru Aoyama, Yukihiko Hiroshima, Takanobu Yamada, Naoto Yamamoto, Takashi Ogata, Hiroyuki Ito, Haruhiko Cho, Manabu Shiozawa, Takaki Yoshikawa, Soichiro Morinaga, Yasushi Rino, Wataru Yasui, Munetaka Masuda, Yohei Miyagi, Takashi Oshima

**Affiliations:** 1 Department of Gastrointestinal Surgery, Kanagawa Cancer Center, Yokohama, Kanagawa 241-8515 Japan; 2 Department of Biostatistics Informatics, Graduate School of Medicine, The University of Tokyo, Tokyo, 113-8658 Japan; 3 Department of Molecular Pathology, Hiroshima University Institute of Biomedical and Health Science, Hiroshima, 734-8551 Japan; 4 Advanced Medical Research Center, Yokohama City University, Yokohama, Kanagawa 236-0004 Japan; 5 Department of Surgery, Yokohama City University, Yokohama, Kanagawa 236-0004 Japan; 6 Department of Thoracic Surgery, Kanagawa Cancer Center, Yokohama, Kanagawa 241-8515 Japan; 7 Department of Surgery, Tokyo Metropolitan Cancer and Infectious Disease Center Komagome Hospital, Bunkyo-ku, Tokyo, 113-8677 Japan; 8 Department of Gastric Surgery, National Cancer Center Hospital, Chuo-ku, Tokyo, 104-0045 Japan; 9 Kanagawa Cancer Center Research Institute, Yokohama, Kanagawa 241-8515 Japan

**Keywords:** Gastric cancer, Gamma-glutamyl hydrolase (GGH), Folylpolyglutamate synthetase (FPGS), S-1, Prognostic factor

## Abstract

**Purpose:**

The enzymes gamma-glutamyl hydrolase (GGH) and folylpolyglutamate synthetase (FPGS) regulate intracellular folate concentrations needed for cell proliferation, DNA synthesis, and repair. High GGH expression affects 5-FU thymidylate synthase (TS) inhibition and is a risk factor for various malignancies. Here, the clinical significance of *GGH* and *FPGS* expression was investigated in Stage II/III gastric cancer patients undergoing postoperative adjuvant chemotherapy with S-1.

**Methods:**

Surgical specimens of cancer tissue and adjacent normal mucosa, obtained from 253 patients with previously untreated gastric cancer, were examined. *GGH* and *FPGS* mRNA expression was measured by qPCR to evaluate their clinicopathological significance in gastric cancer patients after curative resection.

**Results:**

While *FPGS* expression showed no significant differences between the cancerous and normal samples, *GGH* expression was higher in cancer tissue than in adjacent normal mucosa. High *GGH* expression was correlated with age, histological type, and vascular invasion. Overall survival (OS) of patients with high *GGH* mRNA expression was significantly poorer than of patients with low *GGH* expression. Multivariate analysis showed that high GGH expression was an independent prognostic factor of OS (HR: 2.58, 95% CI 1.29–5.16). Patients who received S-1 adjuvant treatment showed a significantly poor OS between high *GGH/*low *FPGS* and low *GGH*/high *FPGS*. Patients without adjuvant treatment showed no significant difference.

**Conclusion:**

*GGH* expression was significantly higher in gastric cancer tissue than in adjacent normal mucosa. High *GGH* and low *FPGS* expression is a useful independent predictor of poor outcomes in stage II/III gastric cancer patients undergoing postoperative adjuvant chemotherapy with S-1.

## Introduction

Gastric cancer was the fifth most common in new cases of cancer (1,033,701 cases) and the third most common in cancer-related deaths (781,631 deaths) in 2018 (Bray et al. 2018). Curative resection and postoperative adjuvant chemotherapy is the standard treatment for locally advanced gastric cancer in Japan. Since Adjuvant Chemotherapy Trial of S-1 for Gastric Cancer (ACTS-GC), adjuvant chemotherapy with S-1 is one of the standard treatments for pathological TNM stage II/III gastric cancer (except pT1N2-3/pT3N0) for the prevention of recurrence (Sakuramoto et al. 2007; Japanese gastric cancer treatment guidelines 2014 (ver. 4) 2017). Moreover, CLASSIC and JACCRO-07 trials have been used to confirm the effectiveness of capecitabine plus oxaliplatin (CapeOX) therapy and S-1 plus docetaxel (DS) therapy; fluoropyrimidine is still the key drug used in the postoperative adjuvant chemotherapy for gastric cancer (Sakuramoto et al. 2007; Bang et al. 2012; Noh et al. 2014). However, the outcomes of treatment are still insufficient, as recurrence occurs in 20–60% of patients, even after a complete resection and the administration of the appropriate adjuvant therapy (Maehara et al. 2000; Rivera et al. 2007). The personalization of postoperative adjuvant chemotherapy treatment using biomarkers is a promising strategy to improve the survival of patients with localized advanced gastric cancer. However, a specific biomarker for use in predicting the therapeutic effects of fluorinated pyrimidine and the long-term outcomes of patients with locally advanced gastric cancer has yet to be identified (Pizzorno et al. 1988; Shubbar et al. 2013; Melling et al. 2017).

Folic acid is a molecule which is necessary for cell proliferation, DNA synthesis, and repair. Gamma-glutamyl hydrolase (GGH) and folylpolyglutamate synthetase (FPGS) are enzymes which regulate intracellular folate concentrations (Bailey 2010). GGH promotes the production of monoglutamyl acid folate, a metabolite of folic acid required for DNA synthesis. On the other hand, FPGS catalyzes the hydrolysis of monoglutamate folate into polyglutamate, which has a high intracellular retention (Bailey 2010). Thus, GGH and FPGS strongly influence DNA synthesis in cancer cells.

Previous studies have reported that high GGH expression is a risk factor for the prognosis of various malignancies (Shubbar et al. 2013; Melling et al. 2017). Moreover, the expression of GGH and FPGS was reported to affect thymidylate synthase (TS) inhibition of 5-fluorouracil (5-FU) in a range of malignancies (Cheradame et al. 1997; Sakamoto et al. 2008; Kim et al. 2013). Recently, it was reported that high GGH expression is a risk factor of lymph node recurrence after surgery in patients with stage II/III gastric cancer, using clinical specimens of ACTS-GC (Terashima et al. 2017). However, the relationship between the expression of GGH and FPGS and the effect of S-1 in adjuvant chemotherapy has not yet been evaluated. In this study, we examined the clinical significance of GGH and FPGS expression in patients with stage II/III gastric cancer who were undertaking postoperative adjuvant chemotherapy with S-1.

## Materials and methods

### Patients and samples

We studied surgical specimens of cancer tissue and adjacent normal mucosa obtained from 253 patients with gastric cancer undergoing gastrectomy as the first treatment. The patients underwent surgery at the Department of Surgery (Yokohama City University), the Gastroenterological Center (Yokohama City Medical Center), and the Department of Gastrointestinal surgery (Kanagawa Cancer Center) between March 2002 and July 2012. Informed consent was obtained from each patient, with all protocols approved by the ethics committees of each institute before the initiation of the study. All tissue samples were embedded in O.C.T. compound (Sakura Finetechnical Co., Ltd., Tokyo) and immediately stored at − 80 °C until further use. None of the patients had any other malignancies. The specimens were stained with hematoxylin and eosin and examined histopathologically. Sections consisting of > 80% cancer cells were used to prepare total RNA.

### Quantitative reverse-transcriptase polymerase chain reaction (qRT-PCR)

qRT-PCR was performed with iQ SYBR Green Supermix (Bio-Rad Laboratories). PCR was carried out in a total volume of 15 μl, which included 0.2 μg of cDNA derived from 75 ng of RNA, 0.4 μM of each primer, 7.5 μl of iQ SYBR Green Supermix containing dATP, dCTP, dGTP, and dTTP at concentrations of 400 μM each, as well as 50 units/ml of iTag DNA polymerase. The PCR cycles consisted of 3 min at 95 °C, followed by 40 cycles of denaturation of the cDNA for 10 s at 95 °C, annealing for 10 s, a primer extension for 20 s at 72 °C, followed by 10 min at 72 °C. The annealing temperature was set at 57 °C and 59 °C for GGH and FPGS, respectively. To distinguish specific from nonspecific products and primer dimmers, melting curve analyses were performed. To evaluate the specific mRNA expression in the samples, a standard curve was produced for each run, measuring three points of the human control cDNA (Clontech Laboratories, Inc., CA, USA). The concentration of each sample was calculated by relating its crossing point to a standard curve. The measurement was performed three times and the average value was adopted. The PCR primer sequences of GGH, FPGS and β-actin, used as an internal control, are shown in Table 1.

**Table 1:** PCR primers and conditions

Gene	Primer	Annealing temperature (°C)	Product size (bp)
*GGH*			
Sense	5′-AGTTGCGTTACACCCTTTCTTGAC-3′	57	151
Antisense	5′-GCTCGCTCCAACCGACTGC-3′		
*FPGS*			
Sense	5′-TTCCGCTTCCTGACACTC-3′	59	116
Antisense	5′-GGCTTCCTGATGATGTTGG-3′		
*β*-*Actin*			
Sense	5′-AGTTGCGTTACACCCTTTCTTGAC-3′	60	171
Antisense	5′-GCTCGCTCCAACCGACTGC-3′		

### Statistical analysis

Gene expression levels were compared between gastric cancer and adjacent normal mucosa by the Wilcoxon test. The expression levels of *GGH* and *FPGS* mRNA were categorized as low or high based on a cut-off value calculated using to the maximum Chi-square (*χ*^2^) test. The relationship between mRNA expression and potential explanatory variables, including age, gender, tumor size, depth of invasion, lymph-node metastasis, distant metastasis, stage, lymphatic invasion, venous invasion, and histological type, were evaluated with the *χ*^2^ test. The associations between the expression of *GGH* and *FPGS* mRNA and survival were assessed using the Kaplan–Meier method, then compared using the log-rank test. A Cox proportional-hazards model was used to perform univariate analyses and stepwise multivariate analyses to determine the risk factors. The software program SPSS (ver. 23.0; IBM Corp., Armonk, NY, USA) was used to perform all the statistical analyses. Two‐sided *P* values were calculated, and *P* < 0.05 was defined as a statistically significant difference. Data were expressed as medians (range).

## Results

### *GGH* and *FPGS* mRNA expression in gastric cancer tissue and adjacent normal mucosa

**Fig. 1:**
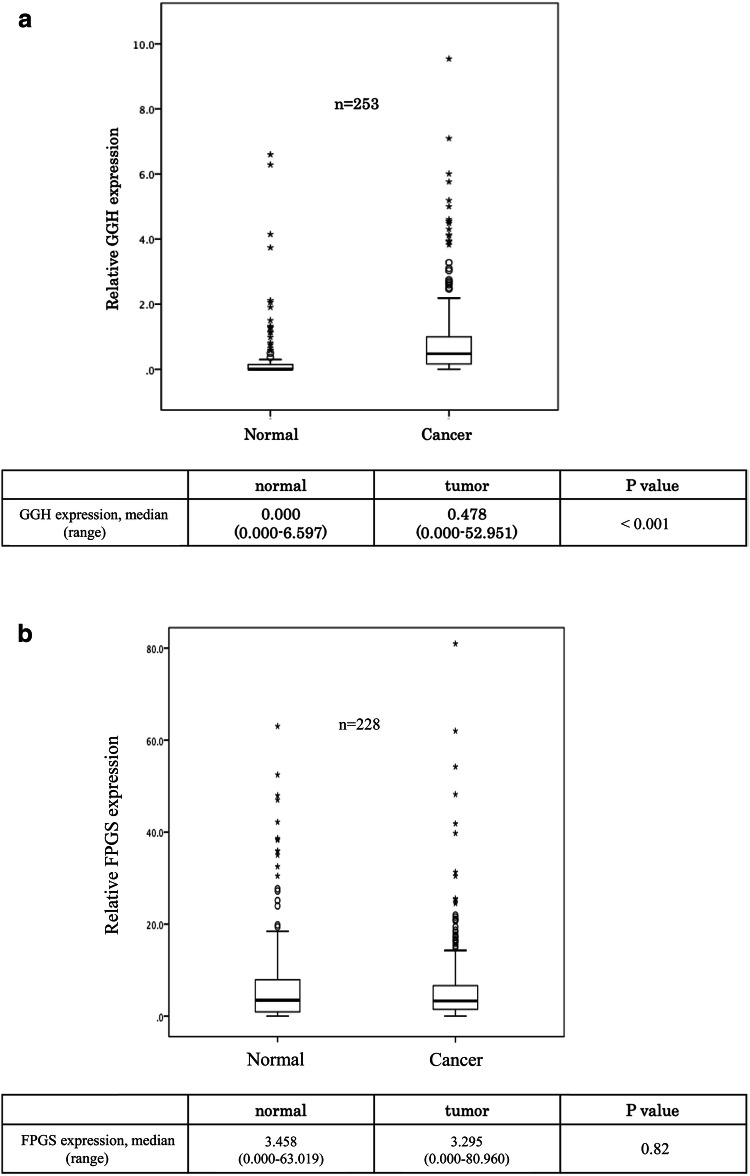
**a** Comparison of expression levels of the GGH gene between gastric cancer tissue and adjacent normal mucosa (*P* < 0.001). **b** Comparison of expression levels of the FPGS gene between gastric cancer tissue and adjacent normal mucosa (*P* = 0.82)

The *GGH* mRNA expression levels were higher in cancer tissue (0.478 [0.000–52.951]) than in adjacent normal mucosa (0.000 [0.000–6.597] *P* < 0.001). On the other hand, there was no significant difference in the *FPGS* mRNA expression level between cancer tissue (3.458 [0.000–63.019]) and adjacent normal mucosa (3.295 [0.000–80.960] *P* = 0.67) (Fig. 1a, b).

### Relationship of *GGH* and *FPGS* mRNA expression levels to clinicopathological features

The expression levels of the *GGH* and *FPGS* genes were categorized as low or high based on a cut-off value calculated using the maximum Chi-square test. The relationship of the expression level of *GGH* mRNA or *FPGS* mRNA to the clinicopathological features was then examined. The expression level of *GGH* mRNA was related to age, histological type, and vascular invasion. On the other hand, the expression level of *FPGS* mRNA was related to vascular invasion and lymphatic invasion (Table 2).

**Table 2:** Comparison of patient’s background and pathological outcomes

Variables	All Patients (*n* = 253)	GGH expression		*P* value	FPGS expression		*P* value
		High group (*n* = 211)	Low group (*n* = 42)		High group (*n* = 222)	Low group (*n* = 31)	
Age (years), median (range)	65 (38–98)	70 (48–98)	64 (38–87)	< 0.001	68 (44–89)	69 (380–98)	0.46
Gender				0.94			0.25
Male	170 (67.2%)	142 (67.3%)	28 66.7%)		152 (68.5%)	18 (58.1%)	
Female	83 (32.8%)	69 (32.7%)	14 (33.3%)		70 (31.5%)	13 41.9%)	
Histological type				0.012			0.66
Undifferentiated	140 (55.3%)	108 (51.2%)	32 76.2%)		124 (55.9%)	16 (51.6%)	
Differentiated	112 (44.3%)	102 48.3%)	10 (23.8%)		98 (44.1%)	15 (48.4%)	
Tumor size (mm)				0.12			0.84
< 65	135 (53.4%)	108 (51.2%)	27 (64.3%)		119 (53.6%)	16 (51.6%)	
65 ≤	118 (46.6%)	103 (48.8%)	15 (35.7%)		103 (46.4%)	15 (48.4%)	
Pathological *T* factor				0.80			0.32
T1–3	119 (47.0%)	100 47.4%)	19 (45.2%)		107 (48.2%)	12 (38.7%)	
T4	134 (53.0%)	111 (52.6%)	23 (54.8%)		115 (51.8%)	19 (61.3%)	
Pathological *N* factor				0.60			0.69
N0	56 (22.1%)	48 (22.75%)	8 (19.1%)		50 (22.5%)	6 (19.4%)	
N1–3	197 (77.9%)	163 (77.3%)	34 (81.0%)		172 (77.5%)	25 (80.6%)	
Stage				0.71			0.59
II	103 (49.7%)	87 (41.2%)	16 (38.1%)		89 (40.1%)	14 (45.2%)	
III	150 (59.3%)	124 (58.8%)	26 (61.9%)		133 (59.9%)	17 (54.8%)	
Vascular invasion				0.028			0.086
v0	73 (28.9%)	55 (26.1%)	18 (42.9%)		60 (27.0%)	13 (41.9%)	
v1–3	180 (71.1%)	156 (73.9%)	24 (57.1%)		162 (73.0%)	18 (58.1%)	
Lymphatic invasion				0.87			0.094
ly0	81 (32.0%)	68 (32.2%)	13 (31.0%)		67 (30.2%)	14 (45.2%)	
ly1–3	172 (68.0%)	143 (67.8%)	29 (69.0%)		155 (69.8%)	17 (54.8%)	
Adjuvant therapy				0.35			0.46
Yes	146 (42.3%)	119 (56.4%)	27 (64.3%)		130 (58.6%)	16 (51.6%)	
No	107 (57.7%)	92 (43.6%)	15 (35.7%)		92 (41.4%)	15 (48.4%)	

### Uni- and multivariate analysis of the relationship of clinicopathological factors and *GGH*/*FPGS* gene expression levels to overall survival

Univariate Cox regression analyses found that high levels of *GGH* mRNA expression, pathological stage, and lymphatic invasion were the significant prognostic factors. On the other hand, according to multivariate Cox regression analysis, high levels of *GGH* mRNA expression, pathological stage, lymphatic invasion, and no postoperative adjuvant treatment with S-1 were the independent prognostic factors. The expression of *FPGS* mRNA was not selected as a significant prognostic factor (Table 3).

**Table 3:** Uni- and multivariate Cox proportional-hazards analysis of clinicopathological factors (overall survival)

Characteristics	Number	Univariate			Multivariate		
		HR	95% CI	*P* value	HR	95% CI	*P* value
Expression of GGH				0.02			0.006
Low	42	1.00			1.00		
High	211	2.27	1.14–4.51		2.65	1.32–5.31	
Expression of FPGS				0.43			
Low	42	1.00					
High	211	1.27	0.71–2.28				
Age(years)				0.81			
< 65	135	1.00					
65 ≤	118	1.05	0.69–1.62				
Gender				0.16			
Female	83	1.00					
Male	170	1.34	0.88–2.15				
Tumor size (mm)				0.84			
< 65	135	1.00					
65 ≤	118	1.04	0.70–1.57				
Histological type				0.82			
Undifferentiated	140	1.00					
Differentiated	113	1.05	070–1.58				
Pathological Stage				<0.001			<0.001
II	103	1.00			1.00		
III	150	2.33	1.47–3.71		2.44	1.50–3.95	
Vascular invasion				0.28			
v0	73	1.00					
v1–3	180	1.29	0.81–2.03				
Lymphatic invasion				0.008			0.011
ly0	81	1.00			1.00		
ly1–3	172	1.91	1.18–3.08		1.94	1.16–3.22	
Adjuvant therapy				0.49			0.018
Yes	146	1.00			1.00		
No	107	1.15	0.77–1.64		1.68	1.09–2.59	

### Comparison of overall survival rates by expression levels of *GGH* or *FPGS* mRNA in gastric cancer tissue

**Fig. 2:**
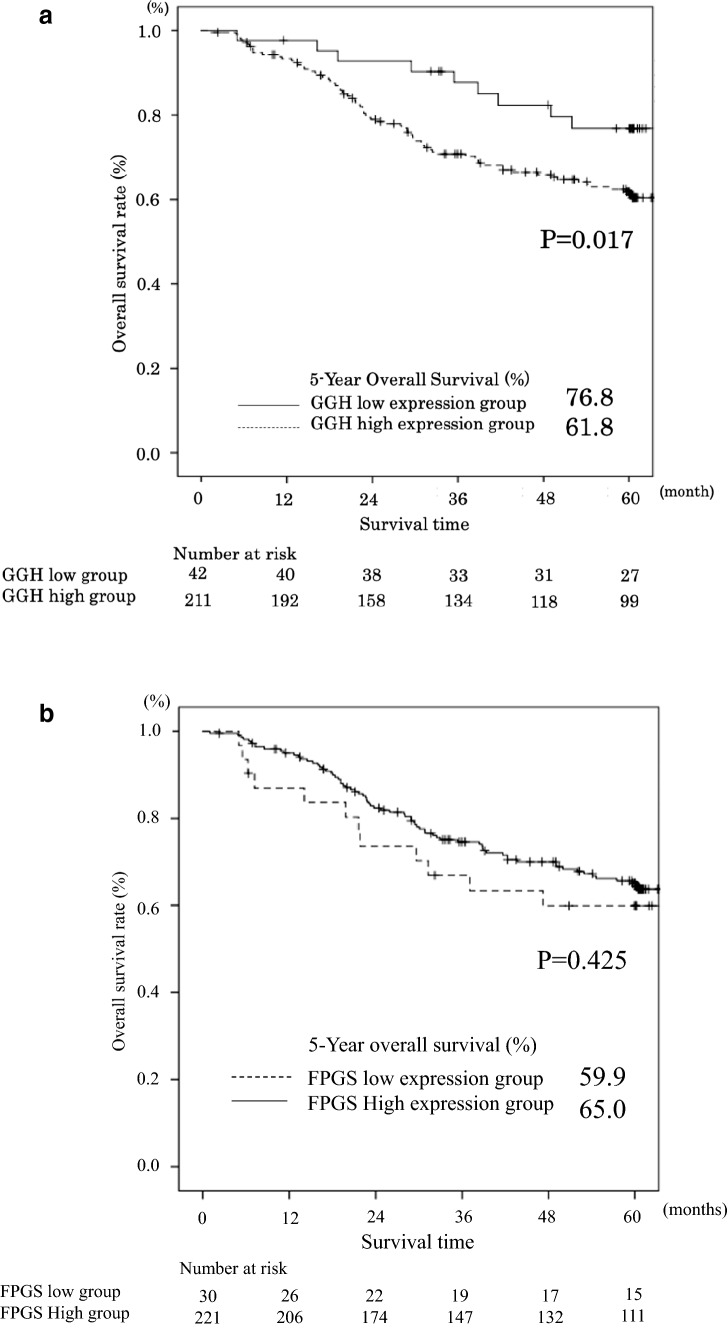
**a** Comparison of overall survival between high and low expression levels of the GGH gene in gastric cancer tissue (*P* = 0.017). **b** Comparison of overall survival between high and low expression levels of the FPGS gene in gastric cancer tissue (*P* = 0.425)

The median follow-up time was 1823 days. During this follow-up period, 127 patients died. The 5-year overall survival rate (OS) in patients with low and high levels of *GGH* mRNA expression were 76.8% and 61.8%, respectively (*P* = 0.017, Fig. 2a). The 5-year OS rate in patients with low and high levels of *FPGS* mRNA expression were 59.9% and 65.0%, respectively (*P* = 0.425, Fig. 2b).

**Fig. 3:**
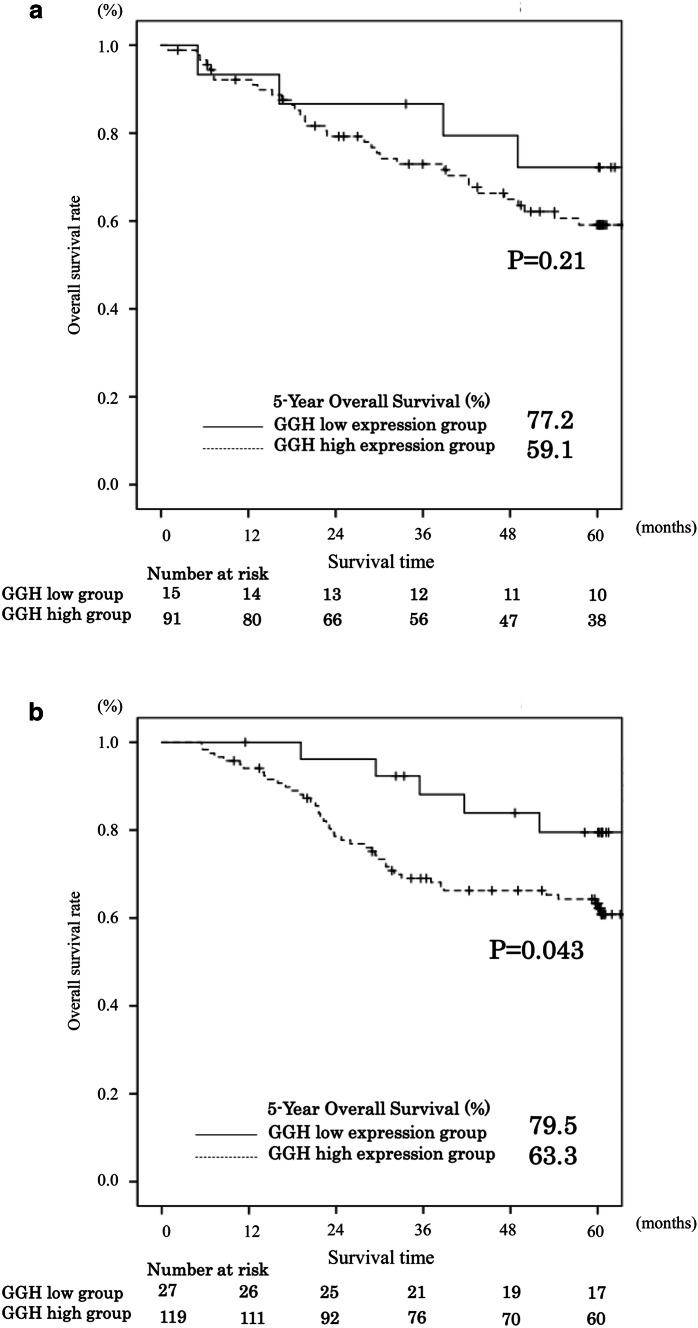
Comparison of overall survival between high and low expression levels of the GGH gene in gastric cancer tissue stratified by whether S-1 adjuvant treatment was performed or not. **a** Patients who did not receive S-1 adjuvant treatment (*P* = 0.21). **b** Patients received S-1 adjuvant treatment (*P* = 0.043)

Stratified by whether S-1 adjuvant treatment was performed or not, the OS in patients who received S-1 adjuvant treatment was significantly poorer in patients with high levels of *GGH* mRNA expression than those with low levels of *GGH* mRNA expression (*P* = 0.043, Fig. 3a). On the other hand, in patients who underwent surgery alone, there was no significant difference in the OS between patients with high and low level of *GGH* mRNA expression (*P* = 0.21, Fig. 3b).

**Fig. 4:**
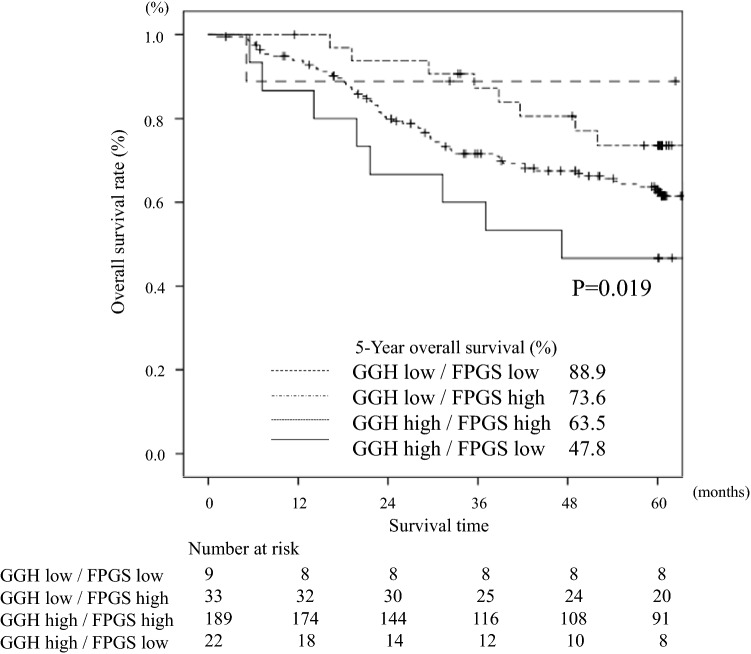
Comparison of overall survival between high and low expression levels of the GGH/FPGS gene in gastric cancer tissue (*P* = 0.019)

**Fig. 5:**
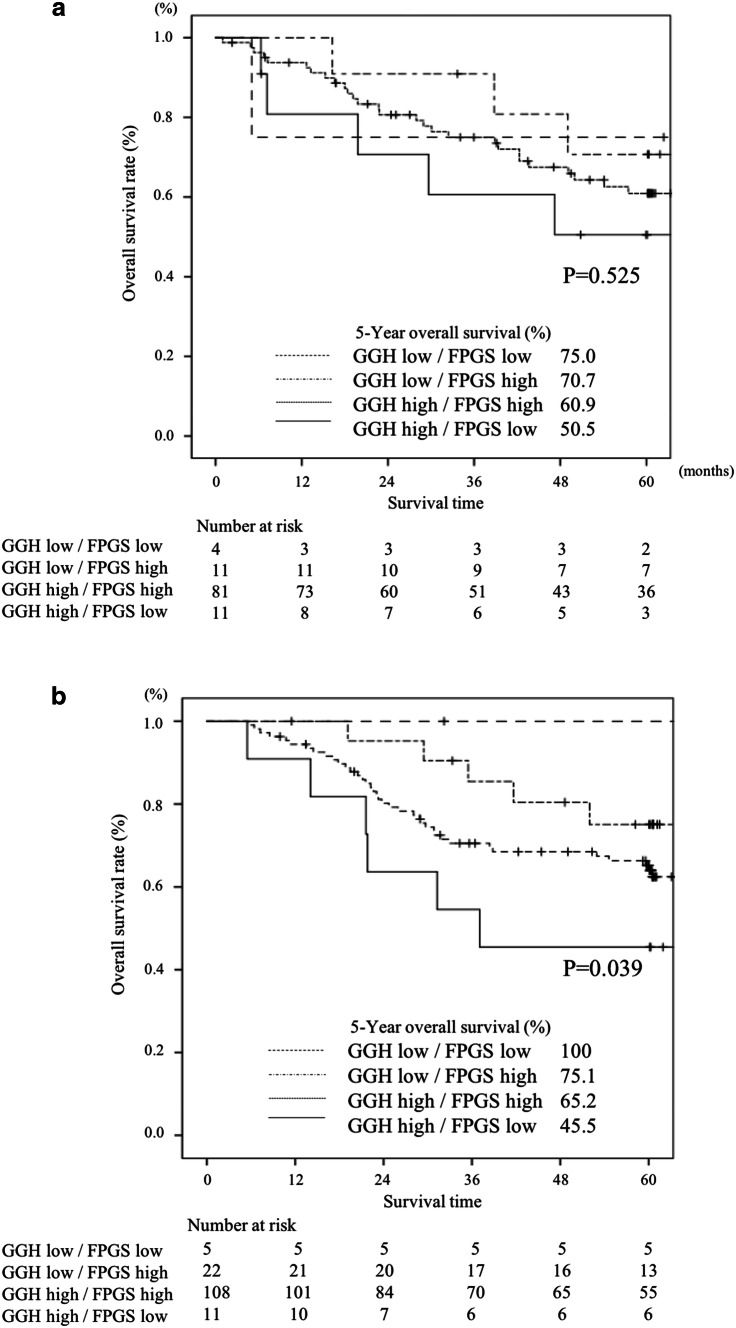
Comparison of overall survival between high and low expression levels of the GGH/FPGS gene in gastric cancer tissue stratified by whether S-1 adjuvant treatment was performed or not. **a** Patients who did not receive S-1 adjuvant treatment (*P* = 0.525). **b** Patients received S-1 adjuvant treatment (*P* = 0.039)

The 5-year OS in patients with various combinations of GGH/FPGS expression, that is, low/low, low/high, high/high, and high/low, was 88.9%, 73.6%, 63.5%, and 47.8%, respectively (*P* = 0.019, Fig. 4). Stratified by whether postoperative adjuvant treatment with S-1 was performed, the OS in patients who received S-1 adjuvant treatment was significantly poorer in patients with high levels of *GGH* mRNA expression and low levels of *FPGS* mRNA expression than those with low levels of *GGH* mRNA expression and high levels of *FPGS* mRNA expression (*P* = 0.039, Fig. 5a). On the other hand, there was no significant difference in patients who underwent surgery alone (*P* = 0.525, Fig. 5b).

## Discussion

In this study, we measured the levels of *GGH* and *FPGS* mRNA expression in cancer tissues and adjacent normal mucosa in patients with stage II/III gastric cancer. We then examined the relationship between the expression levels of these genes and the clinicopathological features and long-term outcomes to evaluate the clinical significance of *GGH* and *FPGS* mRNA expression in gastric cancer tissue in patients with Stage II/III gastric cancer undergoing postoperative adjuvant chemotherapy with S-1.

First, the expression levels of *GGH* and *FPGS* mRNA in gastric cancer tissue and adjacent normal mucosa were compared. Several previous studies have compared the relative expression levels of *GGH* and *FPGS* mRNA between various types of cancer tissue and adjacent normal tissue (Kidd et al. 2005; Pollard et al. 2009; Shubbar et al. 2013). These studies reported that *GGH* mRNA expression was higher in cancer tissue compared to normal tissue in breast cancer (Shubbar et al. 2013) and bladder cancer (Pollard et al. 2009). Our results are consistent with these findings, as the expression levels of *GGH* mRNA were found to be significantly higher in the gastric cancer tissue than in the paired adjacent normal mucosa. As for *FPGS* expression, it has been previously reported that the expression of *FPGS* was higher in cancer tissue than normal mucosa in colorectal cancer (Odin et al. 2003; Kidd et al. 2005). However, in our study, there was no significant difference in the expression levels of *FPGS* mRNA between the cancer tissue and adjacent normal tissue.

Next, the relationship between the expression levels of *GGH* and *FPGS* mRNA and the clinicopathological features in patients with stage II/III gastric cancer were examined. It was reported that GGH expression was significantly associated with a high histological tumor grade (BRE grade III, *P* < 0.001), as well as ER/PR receptors in patients with breast cancer (Shubbar et al. 2013). As for FPGS, to the best of our knowledge, there are no reports. In this study, high levels of *GGH* mRNA expression were significantly correlated with age, histological type, and vascular invasion. High levels of *FPGS* mRNA expression, on the other hand, were found to be related to vascular invasion and lymphatic invasion.

The relationship between the expression levels of *GGH* and *FPGS* mRNA in cancer tissue and long-term outcomes in patients with stage II/III gastric cancer was then assessed. Several studies have previously reported that patients with high levels of *GGH* mRNA expression in cancer tissue resulted in significantly poor outcomes compared to patients with low levels of *GGH* mRNA expression. Melling et al. (2017) reported that 10-year recurrence-free survival was significantly higher in patients with high levels of *GGH* expression compared to patients with low level of *GGH* expression in ERG negative prostate cancer (*P* = 0.0002). Shubber et al. (2013) reported that 8-year disease-specific survival (DSS) was significantly different between patients expressing GGH (39%) and patients whose tumors were GGH-negative (68%, *P* = 0.037) in invasive breast cancer. In addition, univariate analysis showed that GGH expression exhibited a lower DSS probability, with a 2.7-fold increase in risk of death (*P* = 0.04) (Shubbar et al. 2013). As for FPGS, it was reported that 5-year tumor-specific survival (TSS) was significantly better in patients with high levels of *FPGS* mRNA expression (75%) than patients with low levels of *FPGS* mRNA expression (35%, *P* = 0.002). In addition, *FPGS* expression has been previously reported to be an independent significant prognostic factor of TSS in patients with colorectal cancer (Odin et al. 2003).

In present study, the 5-year OS was significantly poorer in patients with high levels of *GGH* mRNA expression than in those with low expression levels. In patients with stage II/III gastric cancer who were administrated adjuvant chemotherapy with S-1, although there was no significant difference in the 5-year OS between patients with high and low levels of *FPGS* mRNA expression, the 5-year OS in patients with the combination of high *GGH* mRNA and low *FPGS* mRNA expression levels in cancer tissue was significantly poorer than that in the other patients.

The elucidation of the mechanism whereby high levels of *GGH* expression and low levels of FPGS expression could be used as prognostic biomarkers in patients with locally advanced gastric cancer for which the administration of postoperative adjuvant chemotherapy with S-1 is not currently sufficient. Previous reports have suggested the following mechanism: 5-FU is an active ingredient of S-1 that inhibits the action of thymidylate synthase (TS) and suppresses DNA synthesis and cell proliferation by forming a trimer with 5-fluorodeoxyuridylate (Fd-UMP), which is a metabolite of 5-FU (Longley et al. 2003; Wilson et al. 2014). The polyglutamate folate is produced by the folate metabolism and induces the formation of the trimer (Moran 1999). GGH promotes the production of monoglutamyl acid folate, a metabolite of folic acid required for DNA synthesis. Meanwhile, FPGS catalyzes the hydrolysis of monoglutamylate folate into polyglutamate, which has a high intracellular retention (Bailey 2010). Thus, high *GGH* and/or low *FPGS* activity reduces 5,10-methylenetetrahydrofolic acid, as well as the TS, which can bind to 5,10-methylenetetrahydrofolic acid. As a result, it is possible that a small amount of 5-FU can exert a TS inhibitory effect in patients with high *GGH* and/or low *FPGS* activity (Moran 1999). These mechanisms indicated that the outcomes of patients with high levels of *GGH* expression and low levels of *FPGS* expression in cancer tissue were poorer than the other patients. Moreover, when combined with our results, these findings suggest that a combination of high *GGH* expression and low *FPGS* expression in cancer tissue could be used as prognostic biomarkers in patients with locally advanced gastric cancer undergoing postoperative adjuvant chemotherapy with S-1 after curative resection.

This study has several limitations. First, the study examined only mRNA expression in gastric cancer tissues. Considering its clinical utility as a biomarker, future studies should examine both mRNA and protein expression levels in the same specimen. Second, there was an issue regarding the heterogeneity of the gastric cancer tissue. The sample from which the mRNA was extracted was a 5-mm square stomach cancer tissue, including the deepest part, which, however, did not completely represent the entire tumor. Third, since *GGH* and *FPGS* have contrasting effects, the prognosis of the high *GGH*/low *FPGS* group could be predicted to be the worst, and, inversely, that of the low *GGH*/high *FPGS* group the best. The present study demonstrated that a significant difference was observed when comparing the OS in the four groups based on different combinations of *GGH* and *FPGS* mRNA expression. In addition, there was an identically significant difference in the OS of the four groups with patients that had received S-1 adjuvant treatment and the analysis of the two groups with high or low GGH. Although the obtained result was different from the hypothesis, which stated that the prognosis of the low *GGH*/high *FPGS* group would be the best, the number of low *GGH*/low *FPGS* patients was nine, which may be the cause of the lack of statistical power. As such, it is possible that the combination of *GGH/FPGS* mRNA may have an enhanced accuracy as a biomarker.

In conclusion, high levels of *GGH* mRNA expression and low levels of *FPGS* mRNA expression in cancer tissue may be useful predictive biomarkers for the survival of patients with stage II/III gastric cancer undergoing postoperative adjuvant chemotherapy with S-1 after radical resection. It would be interesting for future studies to verify the possibility of personalizing treatments by selecting the appropriate regimen based on the expression levels of *GGH* and *FPGS* for patients with locally advanced gastric cancer in a clinical trial.
